# Hepatic arterial infusion chemotherapy versus systemic chemotherapy for advanced intrahepatic cholangiocarcinoma: a meta-analysis of survival outcomes

**DOI:** 10.3389/fimmu.2025.1640970

**Published:** 2025-07-16

**Authors:** Di Zeng, Zhimeng Cheng, Geng Liu, Jiong Lu, Bei Li

**Affiliations:** ^1^ Division of Biliary Surgery, Department of General Surgery, West China Hospital, Sichuan University, Chengdu, Sichuan, China; ^2^ Research Center for Biliary Diseases, West China Hospital, Sichuan University, Chengdu, Sichuan, China

**Keywords:** intrahepatic cholangiocarcinoma (ICC), hepatic arterial infusion chemotherapy (HAIC), survival outcomes, meta-analysis, prognostic factors (PF)

## Abstract

**Background:**

Intrahepatic cholangiocarcinoma (iCC) is an aggressive hepatobiliary malignancy with limited therapeutic options and poor survival outcomes. Hepatic arterial infusion chemotherapy (HAIC) has emerged as a promising treatment alternative to systemic chemotherapy, but its clinical benefits require comprehensive evaluation.

**Methods:**

A systematic review and meta-analysis were conducted, including 10 studies with 1,493 patients. Data on overall survival (OS), progression-free survival (PFS), and key prognostic factors were extracted. Pooled hazard ratios (HR) were calculated using a random-effects model.

**Results:**

HAIC significantly improved OS (HR = 0.51, p < 0.001) and PFS (HR = 0.58, p < 0.001) compared to systemic chemotherapy. Subgroup analyses revealed consistent benefits across various patient characteristics, including age, tumor stage, and baseline liver function. Patients with lower tumor burden (HR = 0.45) and ECOG performance status ≤1 (HR = 0.50) derived the greatest benefit. Additionally, patients with CA 19–9 levels <1,000 U/mL showed significantly improved OS (HR = 0.48).

**Conclusion:**

HAIC prolongs survival and improves disease control in advanced iCC patients compared to systemic chemotherapy. These findings support the adoption of HAIC as a valuable treatment strategy for selected patients, particularly those with lower tumor burden and favorable performance status.

**Systematic review registration:**

https://www.crd.york.ac.uk/PROSPERO/, identifier CRD42024615752.

## 1 Introduction

Intrahepatic cholangiocarcinoma (iCC) is a highly lethal hepatobiliary neoplasm whose incidence is increasing (1). Risk factors for intrahepatic cholangiocarcinoma (iCC) include fibroinflammatory biliary tract diseases such as primary sclerosing cholangitis, Caroli’s disease, hepatolithiasis, and liver fluke infections, as well as systemic conditions like non-alcoholic steatohepatitis and hepatitis (2, 3) Patients with iCC often remain asymptomatic for a long time, leading to late diagnoses when most patients already have advanced, unresectable, or metastatic disease (4). For those who undergo curative-intent resection, the cure rate remains low, with up to 60% experiencing recurrence (5).

The current standard systemic therapy for intrahepatic cholangiocarcinoma (ICC) is platinum-based chemotherapy combined with gemcitabine. The addition of cisplatin to gemcitabine improves median overall survival (OS) from 8.1 months with gemcitabine alone to 11.7 months (6). Similarly, the combination of gemcitabine and oxaliplatin has shown comparable efficacy (7). However, the optimal chemotherapy regimen for patients with advanced BTC refractory to GC has not yet been established (8).

Most patients with advanced ICC present with disease confined to the liver that is unresectable owing to tumor location and/or multifocal involvement (9). A continuous flow of intra-arterial chemotherapy is delivered in the hepatic artery via a surgically implantable subcutaneous pump with a catheter in the gastroduodenal artery (GDA), which is a side branch of the hepatic artery (10). Hepatic arterial infusion chemotherapy (HAIC) delivers high doses of chemotherapy directly to the tumor-rich hepatic artery circulation. Meanwhile, the portal vein maintains the health of the surrounding non-tumorous liver tissue (11). This targeted approach maximizes drug concentration at the tumor site while minimizing systemic toxicity, thanks to the liver’s efficient clearance of chemotherapy through first-pass metabolism (12). Selective hepatic arterial infusion chemotherapy (HAIC) has been used for over four decades and is currently being evaluated for various tumor types and clinical settings (13, 14). In Japan, HAIC has demonstrated effectiveness in treating unresectable hepatocellular carcinoma (15), with studies highlighting its safety and ability to enhance drug delivery to tumors (16).

Hepatic arterial infusion chemotherapy (HAIC) is not widely used as a treatment for intrahepatic cholangiocarcinoma (ICC) worldwide. However, some studies have reported its effectiveness in patients with advanced ICC (17). On the other hand, chemotherapy regimens represented by gemcitabine and platinum-based therapies are more commonly recognized as the standard treatment for ICC in other studies (18, 19). This suggests that the role of HAIC in ICC treatment remains uncertain and requires further investigation. The aim of this meta-analysis is to investigate the therapeutic efficacy of hepatic arterial infusion chemotherapy (HAIC) in the treatment of advanced intrahepatic cholangiocarcinoma (ICC). Specifically, it seeks to determine whether HAIC, compared to traditional systemic chemotherapy regimens, can improve patient survival outcomes. Furthermore, this study aims to identify key prognostic factors that influence survival in ICC patients receiving HAIC and to explore the prognostic characteristics of patients who may derive greater benefit from HAIC than from standard chemotherapy alone.

## 2 Methods

### 2.1 Literature search

This systematic review, registered with the International Prospective Register of Systematic Reviews (PROSPERO) under ID CRD 42024615752, was conducted following the PRISMA (Preferred Reporting Items for Systematic Reviews and Meta-Analyses) guidelines. Two independent reviewers, Zeng D and Wang YQ, carried out a comprehensive literature search across PubMed, Embase, and Web of Science databases, covering all records up to April 2024 and limited to English-language studies. Any disagreements between the reviewers were resolved through discussion with a third reviewer, Wang SF, to reach a consensus.

### 2.2 Inclusion and exclusion criteria

Inclusion Criteria:

1. Studies involving patients diagnosed with advanced intrahepatic cholangiocarcinoma (ICC) through pathological confirmation.
2. Studies evaluating the therapeutic efficacy of hepatic arterial infusion chemotherapy (HAIC) alone or in combination with systemic chemotherapy, specifically in terms of survival outcomes such as overall survival (OS), progression-free survival (PFS), or disease-free survival (DFS).
3. Studies reporting survival data with hazard ratios (HR) and 95% confidence intervals (CI) or providing sufficient statistical data to evaluate the impact of HAIC on survival outcomes.
4. Studies reporting survival data with hazard ratios (HR) and 95% confidence intervals (CI) or providing sufficient statistical data to evaluate the prognostic factors affecting survival outcomes in patients receiving HAIC treatment.
5. Studies focusing on locoregional treatment strategies for ICC, including comparisons between HAIC and standard systemic chemotherapy.

Exclusion Criteria:

1. Studies focusing on other biliary tract malignancies or benign liver conditions, such as extrahepatic cholangiocarcinoma, gallbladder cancer, or hepatocellular carcinoma, without separate data for intrahepatic cholangiocarcinoma.
2. Studies that do not specifically assess the efficacy of HAIC or compare it with traditional systemic chemotherapy in advanced ICC patients.
3. Studies lacking sufficient survival data or essential statistics (e.g., HR, odds ratios [OR], or relative risks [RR]) to evaluate outcomes.
4. Case reports, review articles, conference abstracts, or studies with fewer than ten participants.
5. Studies involving patients without advanced or unresectable ICC, or those focusing solely on surgical or other non-chemotherapy-based treatments.

### 2.3 Statistical analysis

Survival data were analyzed using multivariate regression techniques, with hazard ratios (HRs) and their corresponding 95% confidence intervals (CIs) as the primary measures. Categorical variables were assessed through odds ratios (ORs). Statistical heterogeneity among studies was evaluated using Cochrane’s Q-test and I² statistics, categorizing heterogeneity as low, moderate, or high at thresholds of 25%, 50%, and 75%, respectively. A random-effects model was applied consistently to account for variability across studies, regardless of the heterogeneity level.

To evaluate publication bias, funnel plots were used for visual inspection of asymmetry, which may suggest bias in study selection or reporting. This was further supported by Egger’s test, a statistical method used to quantify and confirm the presence of publication bias. A significant result (P < 0.05) from Egger’s test would indicate potential bias, requiring cautious interpretation of the pooled results.

To assess the robustness of the findings, sensitivity analyses were conducted by sequentially excluding each study and reanalyzing the remaining data. This approach helped evaluate whether any single study had a disproportionate impact on the overall estimates. These steps ensured that the conclusions were stable and not overly influenced by individual datasets. A p-value < 0.05 (two-tailed) was considered statistically significant.

### 2.4 Quality assessment of studies

The quality of the included studies was assessed independently by two investigators, Zeng D and Wang SF, using the Newcastle-Ottawa Scale (NOS). This tool evaluates studies across three main domains: selection of study groups, comparability of cohorts, and outcome assessment. Each study was assigned a score out of nine, with a score of six or higher indicating acceptable quality for inclusion. Discrepancies between the investigators were resolved through discussion or consultation with a third reviewer to ensure consistency and reliability in the quality assessment. Detailed NOS scoring results are provided in **Supplementary Table 1**.

## 3 Results

### 3.1 Literature search

**Figure 1:**
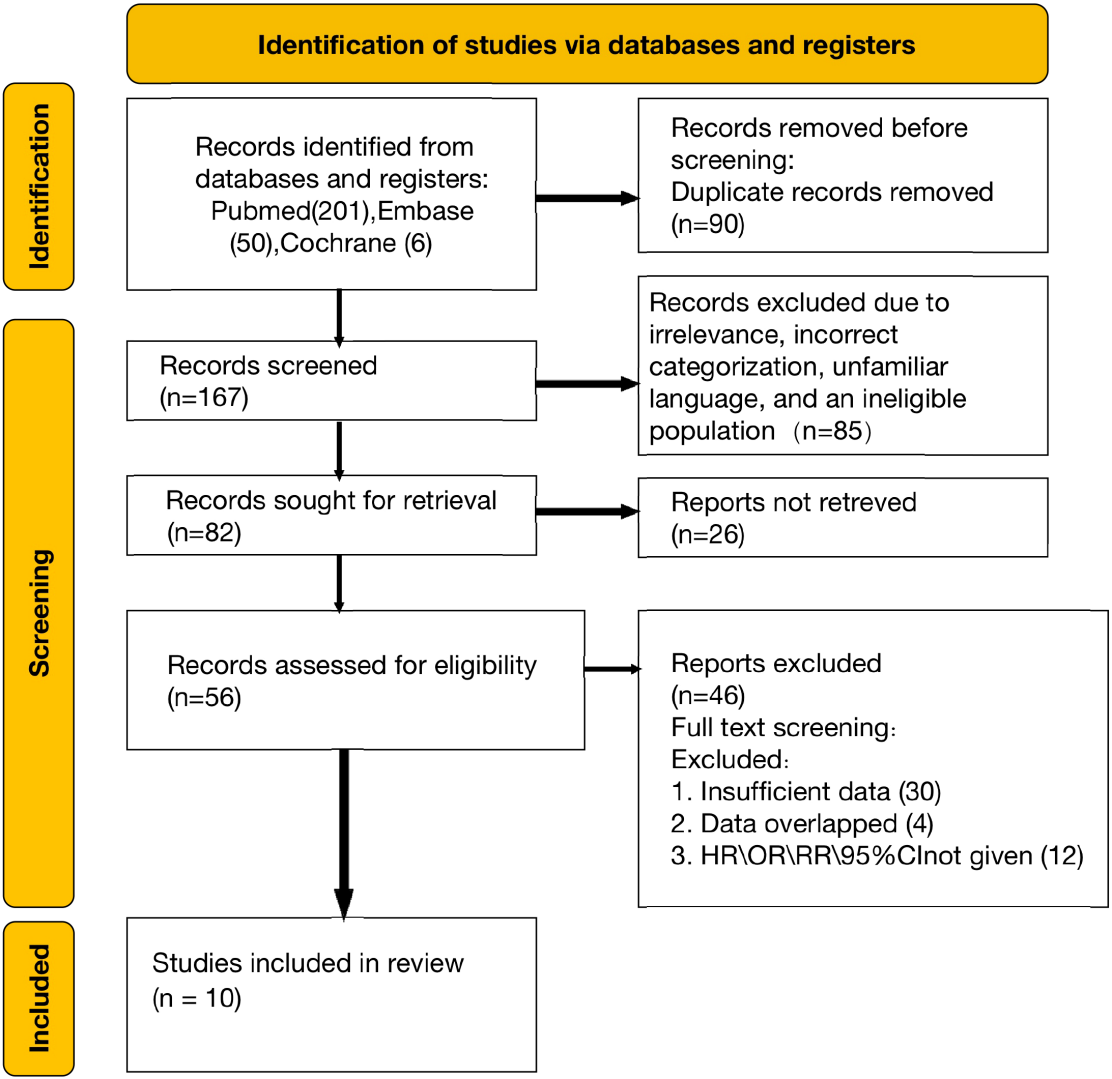
PRISMA flowchart illustrating the study selection process.

A total of 257 articles were initially retrieved from electronic databases, including PubMed, Embase, and Web of Science. After the removal of duplicates and screening for relevance, 167 full-text articles were reviewed for eligibility. Upon detailed evaluation, 10 studies satisfied the inclusion criteria and were incorporated into the qualitative analysis (8, 10, 20–27). The selection process is depicted in the PRISMA flowchart (**Figure 1**).

### 3.2 Study characteristics and quality assessment

This meta-analysis included a total of 1493 patients diagnosed with intrahepatic cholangiocarcinoma (ICC) from studies published between 2018 and 2024. Among the 10 included studies, all compared the effects of hepatic arterial infusion chemotherapy (HAIC) versus systemic chemotherapy on patient survival outcomes. Five studies focused on identifying prognostic factors that influence outcomes in patients treated with HAIC. Additionally, three studies analyzed which prognostic factors determined greater benefit from HAIC compared to chemotherapy, or vice versa. Among the 10 included studies, the Newcastle-Ottawa Scale (NOS) quality assessment demonstrated that most were of high quality, with nine achieving a score of seven or higher (detailed NOS evaluations are available in **Supplementary Table 1**). The PRISMA diagram (**Figure 1**) provides an overview of the study selection process, while the detailed characteristics of these studies are summarized in **Table 1**.

**Table 1:** Summary of the characteristics of included studies.

Study	Year	Country	Number of patients	Treatment in details	Follow up data	Survival information
Zhipeng Lin (21)	2024	China	90	In the HAIC group, patients were treated with hepatic arterial infusion chemotherapy (HAIC) combined with lenvatinib and a PD-1 inhibitor. In the systemic chemotherapy group, patients received first-line treatment with a combination of cisplatin and gemcitabine.	The HAIC+L+P group had a median follow-up duration of 32.8 months, while the systemic chemotherapy group had 22.6 months.	The HAIC+L+P group had a median overall survival (OS) of 16.8 months, while the SC group had a median OS of 11.0 months. The median progression-free survival (PFS) in the HAIC+L+P group was 12.0 months, compared to 6.9 months in the SC group.
Wright (27)	2018	USA	116	In the HAIC group, 12 patients received systemic chemotherapy alongside the pump therapy, with 8 patients (66.7%) receiving both treatments as part of their initial regimen. The group had a median of 5.5 treatments (range 1–14), while two patients in the SIRT-Y90 group received one and three treatments, respectively. In the TACE group, 41 patients received a median of three treatments (range 1–15). The chemotherapy regimens used included 5-fluorouracil (19.5%), cisplatin (9.8%), irinotecan drug-eluting beads (4.9%), and cisplatin/doxorubicin/mitomycin C (2.1%). Additionally, in the surgical resection group, 23.3% of patients (10 out of 43) received preoperative chemotherapy.	This study was designed as a retrospective review of consecutive cases of intrahepatic cholangiocarcinoma (ICC) diagnosed between January 2004 and June 2016. Consecutive patient data were gathered for all patients with ICC from the time of diagnosis through death or the date of last follow-up. The specific follow-up information is unclear.	The HAIC group had a median overall survival of 39 months (95% CI: 32.7–51.3), progression-free survival of 9 months (95% CI: 6.4–11.6), and hepatic progression-free survival of 13 months (95% CI: 6.7–19.3). The TACE group showed a median overall survival of 15 months (95% CI: 11.4–18.6), progression-free survival of 3 months (95% CI: 2.1–3.9), and hepatic progression-free survival of 3 months (95% CI: 2.1–3.9).
Yang (22)	2023	China	146	The HAIC group received hepatic arterial infusion chemotherapy (oxaliplatin 130 mg/m², leucovorin 400 mg/m², and fluorouracil 400 mg/m² bolus followed by 2400 mg/m² continuous infusion over 23–46 hours) every 3 weeks, vs. the SC group, which underwent systemic chemotherapy using either the GEMCIS regimen (cisplatin 25 mg/m² and gemcitabine 1000 mg/m² on days 1 and 8 every 3 weeks) or the GEMOX regimen (oxaliplatin 85 mg/m² on day 1 and gemcitabine 1000 mg/m² on days 1 and 8 every 3 weeks). Both groups could include PD-1 inhibitors or tyrosine kinase inhibitors as needed.	The median follow-up duration was 16.8 months for the HAIC group and 17.7 months for the systemic chemotherapy (SC) group.	Median OS: 18.0 months in the HAIC group vs. 17.8 months in the SC group. Median PFS: 10.8 months in the HAIC group vs. 11.4 months in the SC group. Median IPFS: 13.7 months in the HAIC group vs. 11.4 months in the SC group.
Jiang (23)	2024	China	118	Patients in this study received either systemic chemotherapy (SYS) alone or transarterial chemoembolization (TACE) followed by systemic chemotherapy (TACE+SYS). The treatment approach was discussed in a multi-disciplinary team meeting, where the decision was made according to each patient’s condition. In the TACE+SYS group, patients received TACE first, followed by SYS treatment one week later, with SYS being repeated every 21 days. In the SYS alone group, patients received gemcitabine combined with cisplatin, with cycles repeated at 21-day intervals. Other systemic regimens included gemcitabine with tegafur, capecitabine with oxaliplatin, and other combinations based on patient-specific considerations.	The follow-up for this study was conducted through telephone interviews to assess overall survival (OS), defined as the time from the initiation of treatment to death from any cause or the date of the last follow-up, which was May 17, 2023. Tumor response was evaluated by contrast-enhanced CT or dynamic contrast-enhanced MRI, with the first follow-up recommended 3 months after treatment	Before propensity score matching (PSM), the median overall survival (OS) was 11.3 months (range 9.1–17.8) in the TACE plus systemic chemotherapy (TACE+SYS) group, and 6.4 months (range 5.3–8.4) in the systemic chemotherapy (SYS) alone group. After PSM, the TACE+SYS group continued to show a significantly longer median OS compared to the SYS alone group (P<0.05). The median progression-free survival (PFS) in the TACE+SYS group was 12.0 months, while it was 6.9 months in the SYS alone group.
Franssen (10)	2024	Netherlands		76 received gemcitabine-cisplatin (Gem-Cis) chemotherapy, while 192 patients received hepatic arterial infusion chemotherapy (HAIC). Most patients in the HAIC group (69.8%) were treated with HAIC as a first-line therapy, while 30.2% had previous systemic chemotherapy. HAIC was combined with systemic chemotherapy in 71.9% of patients, with treatment regimens including gemcitabine plus cisplatin or oxaliplatin, gemcitabine monotherapy, and irinotecan. A smaller group of 42 patients (21.9%) received HAIC as first-line treatment without concurrent systemic chemotherapy.	Patients were followed until death or the date they were lost to follow-up. The follow-up data were retrieved from existing medical records and included the date of death or last follow-up. Tumor progression and treatment responses were assessed based on available medical records, but second-line systemic treatment data were not collected. For patients lost to follow-up, the data were censored. Regular follow-up was conducted to monitor the patient's condition after systemic gem-cis treatment or HAIP chemotherapy	Among patients with liver-confined unresectable intrahepatic cholangiocarcinoma (iCCA), those treated with hepatic arterial infusion chemotherapy (HAIC) showed a median overall survival of 27.7 months, with three- and five-year survival rates of 34.3% and 15.1%, respectively. First-line HAIC therapy achieved a median survival of 27.2 months, while second-line therapy reached 30.0 months. For patients receiving HAIC combined with systemic chemotherapy, median survival was 26.4 months, compared to 29.4 months for HAIC alone. In comparison, patients receiving gemcitabine-cisplatin therapy had a median survival of 11.8 months, with a three-year survival rate of 3.5% and no five-year survivors. These findings emphasize the survival advantage of HAIC therapy across different clinical applications.
Zheng (24)	2024	China	202	The triple combination therapy group received FOLFOX-HAIC (hepatic arterial infusion chemotherapy), targeted therapy (TKIs), and immunotherapy (anti-PD-L1/PD-1). The standard chemotherapy (SC) group was treated with either the GemCis or GemOx regimen. The GemCis regimen consisted of gemcitabine and cisplatin, while the GemOx regimen included oxaliplatin and gemcitabine. Both groups might also receive additional therapies based on multidisciplinary review.	The follow-up for patients in this study ranged from March 2015 to June 2023, focusing on those with advanced intrahepatic cholangiocarcinoma (iCCA) who received either the triple combination therapy or standard chemotherapy.	In the triple combination therapy group, the median overall survival (OS) was 20.77 months, the median progression-free survival (PFS) was 9.07 months, and the median event-free progression survival (EPFS) was 11.37 months. The median inter-progression-free survival (IPFS) was 11.03 months. In the standard chemotherapy group, the median OS was 14.83 months, the median PFS was 6.23 months, the median IPFS was 6.73 months, and the median EPFS was 7.13 months. The objective response rate (ORR) in the triple combination therapy group was 35.5%, while in the standard chemotherapy group, it was 14.5%. The disease control rate (DCR) was 77.6% in the triple combination therapy group and 63.2% in the standard chemotherapy group.
Masatsugu Ishii (8)	2022	Japan	34	Patients in the HAIC group received chemotherapy via hepatic arterial infusion, which directly targets the liver, delivering drugs such as gemcitabine, cisplatin, and 5-fluorouracil to the tumors in the liver more effectively. In contrast, patients in the standard chemotherapy group received systemic chemotherapy through intravenous infusion, which circulates the drugs throughout the entire body, including the liver. The HAIC group also included patients who had not previously received chemotherapy, whereas the standard chemotherapy group primarily consisted of patients whose tumors were resistant to earlier systemic treatments. This difference in treatment methods is intended to provide a more focused approach for liver cancers while addressing chemotherapy resistance in the standard group.	The median follow-up period in the patients was 8.3 months (range: 2–36 months) after treat	In the HAIC group, the median overall survival (OS) was 19.7 months, with a six-month OS rate of 67.5% and a one-year rate of 40.5%. The median OS after being determined as gemcitabine plus cisplatin refractory was 6.3 months. In contrast, the standard chemotherapy group had a median OS of 10.8 months, with a six-month OS rate of 30.6%and a zero one-year survival rate.
Yan-Song Lin (25)	2024	China	141	The SC group received the GEMCIS regimen (cisplatin + gemcitabine), the SCP group received the same GEMCIS regimen combined with PD-(L)1 inhibitors, and the HAIC group underwent Hepatic Arterial Infusion Chemotherapy (HAIC) with a FOLFOX regimen, alongside PD-(L)1 inhibitors and lenvatinib. The treatments were administered over 3-week cycles, with variations in the inclusion of immune checkpoint inhibitors and targeted therapies in the latter two groups, and the use of HAIC in the HLP group.	Patients were followed until death or the date of their last follow-up, with the follow-up deadline set to December 31, 2023. The survival status of all patients was updated to this date. Tumor responses were evaluated through computed tomography (CT) and magnetic resonance imaging (MRI) three months after the initiation of treatment, following the RECIST 1.1 criteria. The median follow-up durations across the three treatment groups—HAIC (HLP), SCP, and SC—were 15.5 months, 10.7 months, and 13.0 months, respectively. Progression-free survival (PFS) and overall survival (OS) rates were also calculated. Disease progression and survival status were recorded, with patients who were lost to follow-up being censored	The median progression-free survival (PFS) times were 30.0 months for the HLP group, 10.2 months for the SCP group, and 6.5 months for the SC group. The median overall survival (OS) for the HLP and SCP groups was not reached, while the SC group had a median OS of 21.8 months. The objective response rate (ORR) was 50.0% for the HLP group, 18.4% for the SCP group, and 6.0% for the SC group. The disease control rate (DCR) was 88.1% for the HLP group, 73.5% for the SCP group, and 52.0% for the SC group.
Cai (26)	2021	China	121	In the Hepatic Arterial Infusion Chemotherapy (HAIC) group, patients received 85 mg/m² oxaliplatin, 400 mg/m² leucovorin, and 400 mg/m² 5-FU on day 1, followed by continuous infusion of 2400 mg/m² 5-FU over 46 hours. The treatment was delivered via a microcatheter in the tumor’s blood supply artery, with 6-8 cycles lasting 21 days. In the Transarterial Chemoembolization (TACE) group, chemotherapeutics (epirubicin, mitomycin, and carboplatin) were infused into the tumor’s blood supply artery, followed by embolization with iodized oil (3-25 ml), based on the tumor's characteristics.	The median follow-up time for the entire cohort was 8.4 months (range 0.8–47.2 months). During this period, 14 patients (24.6%) in the Hepatic Arterial Infusion Chemotherapy (HAIC) group and 26 patients (37.7%) in the Transarterial Chemoembolization (TACE) group died.	The median overall survival (OS) time was 19.6 months for the Hepatic Arterial Infusion Chemotherapy (HAIC) group and 10.8 months for the Transarterial Chemoembolization (TACE) group. The 1-year OS rates were 60.1% for HAIC and 38.6% for TACE, while the 2-year OS rates were 42.9% for HAIC and 29.4% for TACE. The median progression-free survival (PFS) was 3.9 months for the HAIC group and 3.7 months for the TACE group, with no significant difference. For overall intrahepatic progression-free survival (OIPFS), the HAIC group had a median of 9.2 months, significantly longer than the TACE group's 4.4 months.
Konstantinidis	2017	USA	525	Patients with liver-confined disease were treated with a combination of hepatic arterial infusion (HAI) and systemic chemotherapy (SYS) in 75% of cases, and with SYS alone in 25%. For patients with regional nodal disease, 76% received only systemic chemotherapy. A subset of patients who were initially considered unresectable underwent resection after conversion chemotherapy, with 4 receiving SYS alone and 4 receiving a combination of SYS and HAI.	At the time of analysis, 79% of the 236 patients had succumbed to the disease. The median overall survival for the entire cohort was 20.1 months (range: 1.3–120.3 months). Among these patients, those with liver-only disease had a median survival of 24.1 months (range: 4–120.3 months), which was longer compared to patients with nodal involvement (17.1 months; range: 1.4–58.9 months) or distant metastases (12.4 months; range: 1.3–59.2 months), regardless of treatment.	Patients treated with combined hepatic arterial infusion chemotherapy (HAIC) and systemic chemotherapy (SYS) achieved a median overall survival of 30.8 months, which slightly decreased to 29.6 months when including those with portal lymph node disease. Additionally, eight patients with initially unresectable tumors responded sufficiently to undergo complete resection, reaching a median survival of 37 months (range: 10.4–92.3 months). Patients receiving systemic chemotherapy (SYS) alone had a median overall survival of 18.4 months, which dropped to 15.9 months when patients with portal lymph node disease were included.

### 3.3 HAIC vs systemic chemotherapy: impact on overall survival in advanced intrahepatic cholangiocarcinoma

**Figure 2:**
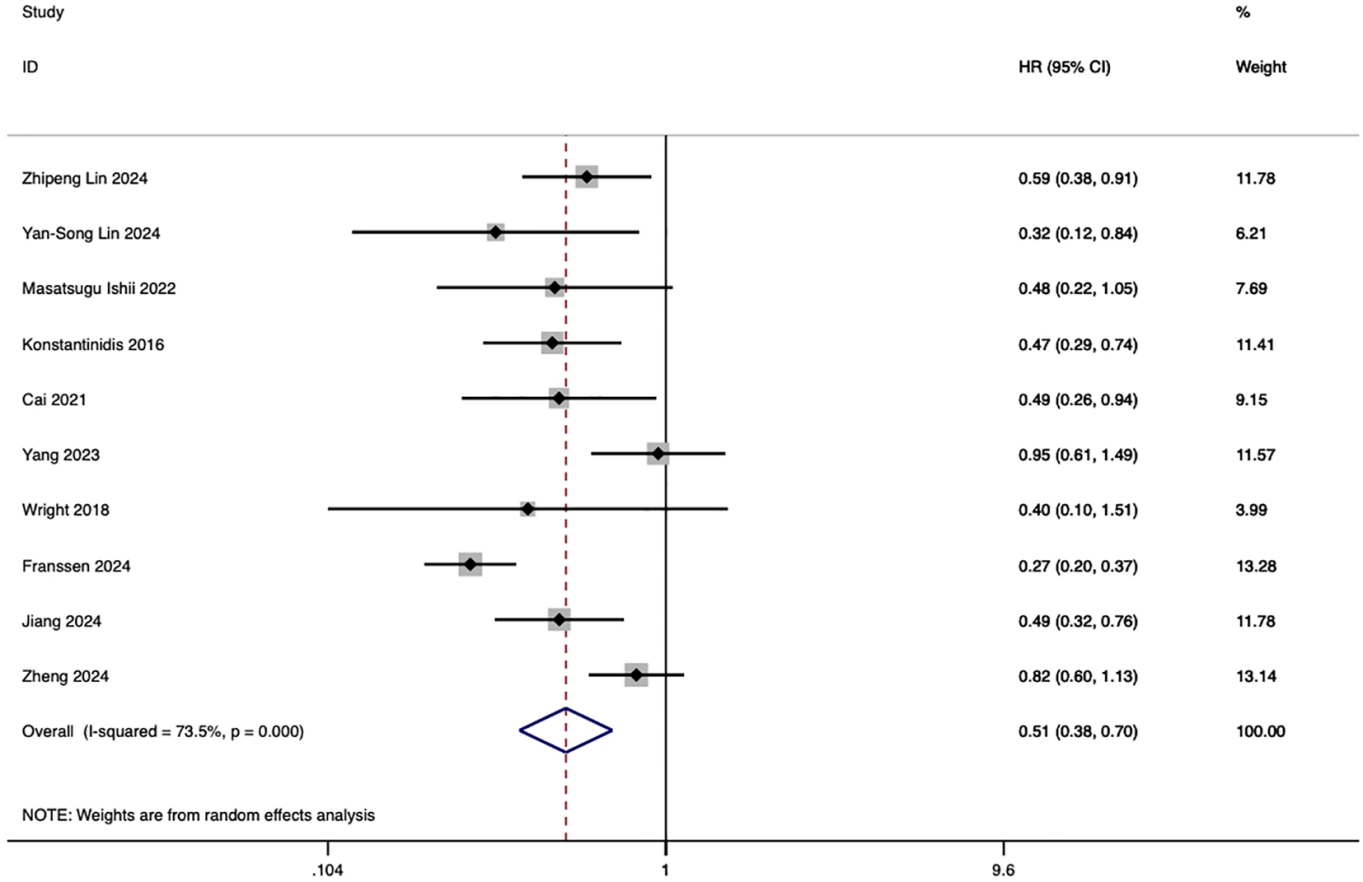
Forest plot of overall survival comparing HAIC and systemic chemotherapy in advanced intrahepatic cholangiocarcinoma (ICC).

Ten studies evaluated the comparative impact of HAIC and systemic chemotherapy on overall survival in advanced intrahepatic cholangiocarcinoma. The pooled analysis of these studies demonstrated that HAIC significantly improved overall survival, with a hazard ratio (HR) of 0.51 (95% CI: 0.38–0.70, p < 0.001) (**Figure 2**).

### 3.4 HAIC vs systemic chemotherapy: impact on progression-free survival in advanced intrahepatic cholangiocarcinoma

**Figure 3:**
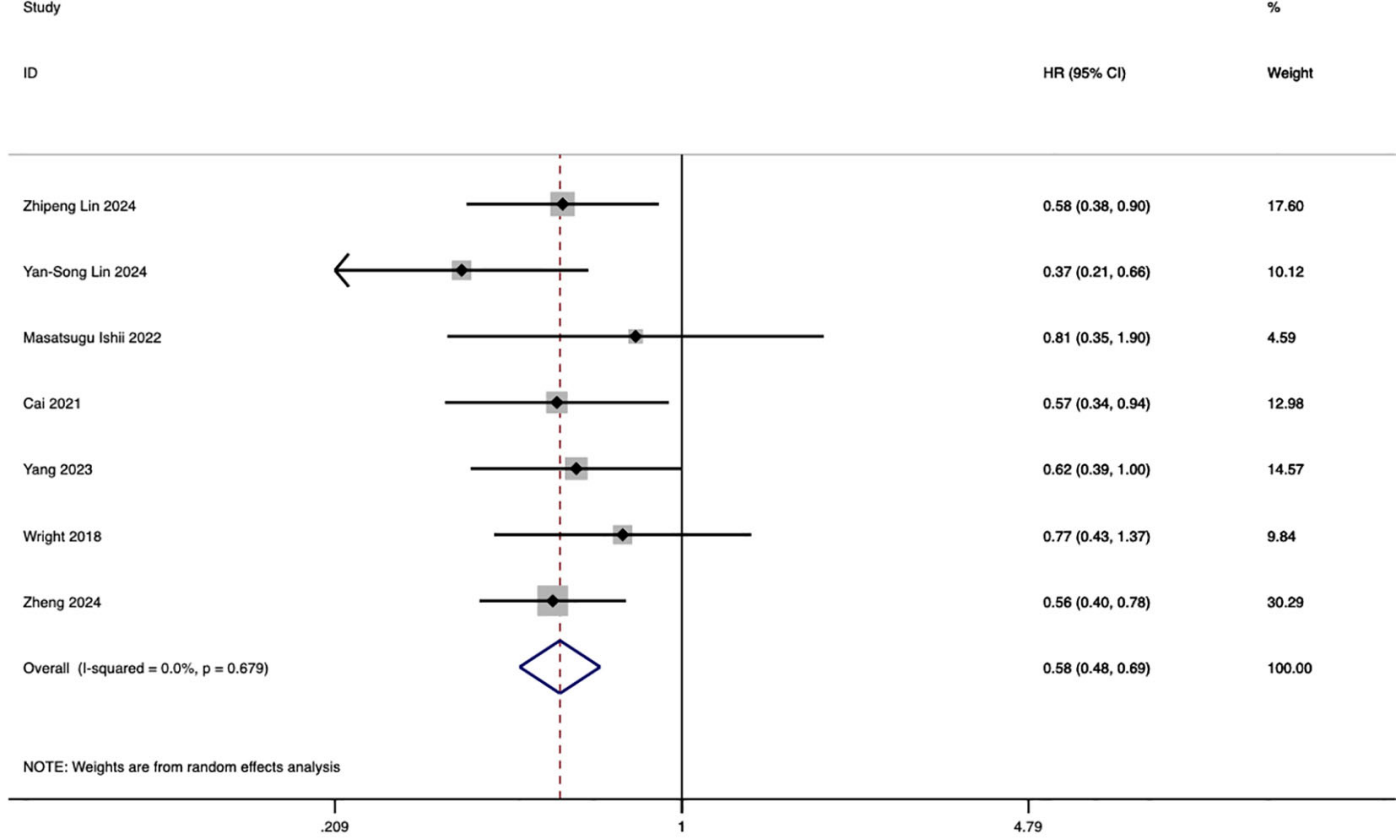
Forest plot of progression-free survival comparing HAIC and systemic chemotherapy in advanced ICC.

Seven studies investigated the comparative impact of HAIC and systemic chemotherapy on progression-free survival in advanced intrahepatic cholangiocarcinoma. The pooled analysis demonstrated that HAIC significantly improved overall survival, with a hazard ratio (HR) of 0.58 (95% CI: 0.48, 0.69; p < 0.001) (**Figure 3**).

### 3.5 Prognostic factors influencing survival in intrahepatic cholangiocarcinoma patients undergoing HAIC therapy

In five studies analyzing prognostic factors influencing survival in intrahepatic cholangiocarcinoma patients undergoing hepatic arterial infusion chemotherapy (HAIC), a total of 15 factors were evaluated for their potential impact on survival outcomes. These factors included age (≥50/60 years), gender (male vs. female), Eastern Cooperative Oncology Group (ECOG) performance status (≥1), multifocal tumor (yes vs. no), tumor location (bilobar vs. unilobar), largest tumor size (>5/10 cm), carbohydrate antigen 19-9 (CA 19-9) levels (elevated levels), CEA levels (>5 ng/mL), TNM stage (stage ≥3), vascular invasion (yes vs. no), lymph node metastasis (yes vs. no), Child-Pugh class (B vs. A), albumin (ALB) levels (<35 g/L vs. >35 g/L), and HBV status (positive vs. negative).

**Figure 4:**
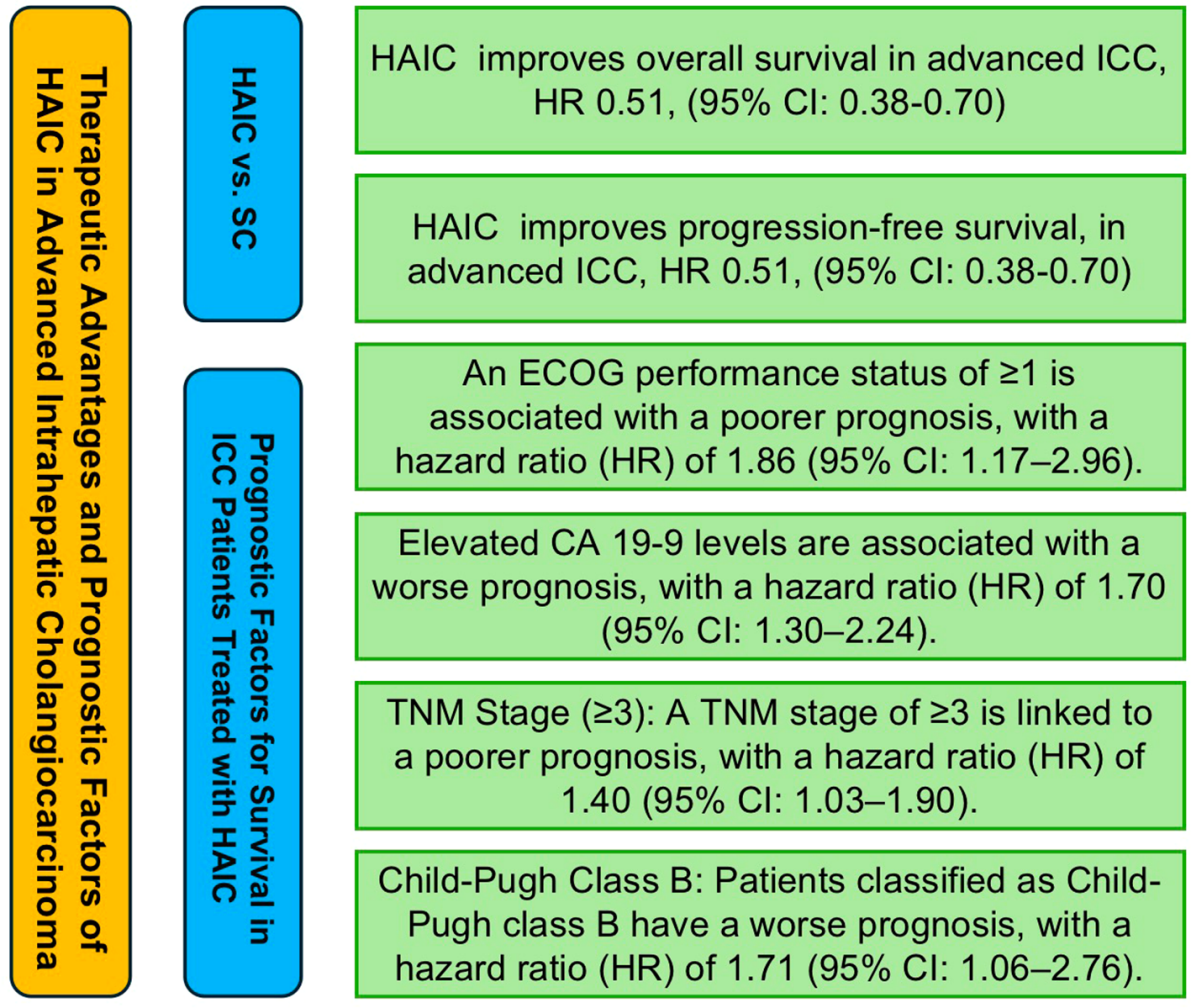
Prognostic factors influencing survival outcomes in ICC patients treated with HAIC.

Among these factors, age (≥50/60 years), gender (male), ECOG performance status (≥1), multifocal tumor (yes vs. no), CA 19–9 levels (elevated), CEA levels (>5 ng/mL), TNM stage (stage ≥3), vascular invasion (yes vs. no), lymph node metastasis (yes vs. no), Child-Pugh class (B vs. A), albumin levels (<35 g/L), and HBV status (positive vs. negative) all demonstrated a negative association with prognosis, although some analyses did not show full statistical significance. Specifically, gender (male) had a hazard ratio (HR) of 1.49 (95% CI: 1.18, 1.88), ECOG performance status (≥1) had an HR of 1.86 (95% CI: 1.17, 2.96), CA 19–9 levels (elevated) had an HR of 1.70 (95% CI: 1.30, 2.24), TNM stage (stage ≥3) had an HR of 1.40 (95% CI: 1.03, 1.90), and Child-Pugh class (B vs. A) had an HR of 1.71 (95% CI: 1.06, 2.76), all of which were significantly associated with poorer prognosis. The results of the above analysis are presented in **Figure 4**.

### 3.6 Prognostic factors influencing the differences between HAIC and systemic chemotherapy in advanced intrahepatic cholangiocarcinoma patients

**Figure 5:**
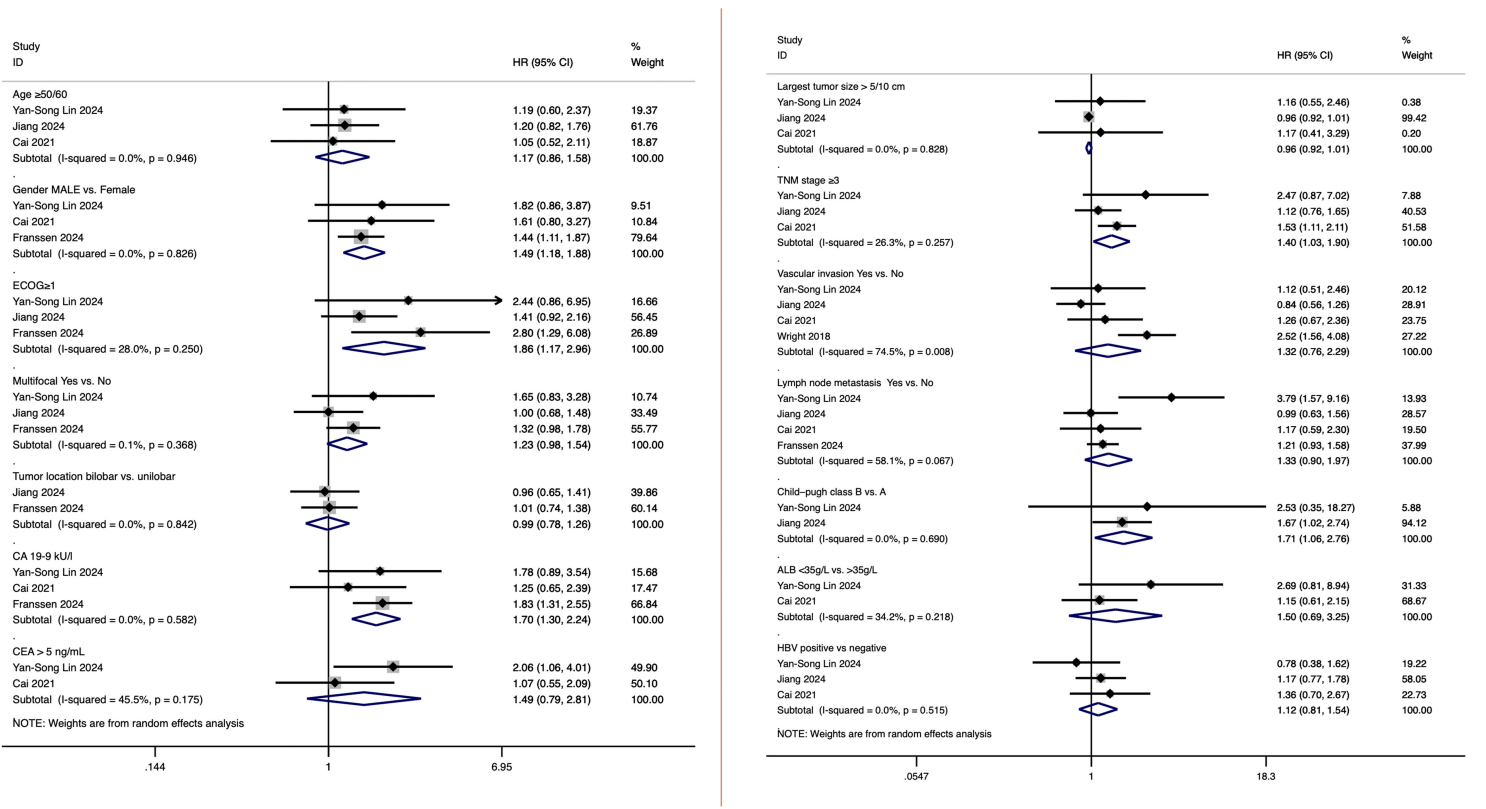
Subgroup analysis of prognostic factors influencing the comparative efficacy of HAIC versus systemic chemotherapy in advanced ICC.

Three original studies evaluated 24 prognostic factors that may influence the differences in survival outcomes between hepatic arterial infusion chemotherapy (HAIC) and systemic chemotherapy in patients with advanced intrahepatic cholangiocarcinoma. These factors included age (>60 vs. ≤60), gender (male vs. female), ECOG performance status (0 vs. ≥1), Child-Pugh class (A vs. B), CEA levels (<5 vs. ≥5 ng/mL), CA 19–9 levels (<40/100 vs. ≥40/100), lymph node metastasis (present vs. absent), distant metastasis (present vs. absent), TNM stage (<3 vs. ≥3), tumor size (<10 cm vs. ≥10 cm), portal vein invasion (present vs. absent), and liver cirrhosis (present vs. absent). These factors were systematically analyzed to identify their potential impact on the efficacy of the two treatments. In nearly all prognostic factor subgroups, hepatic arterial infusion chemotherapy (HAIC) demonstrated better efficacy compared to traditional systemic chemotherapy in advanced intrahepatic cholangiocarcinoma patients. The only exception was observed in patients with CA 19–9 levels <40/100, where HAIC showed slightly worse outcomes with a hazard ratio (HR) of 1.14 (95% CI: 0.66–2.03). Notably, HAIC achieved statistically significant better outcomes in specific subgroups, including patients aged >60 (HR: 0.58, 95% CI: 0.41–0.82), those with an ECOG performance status of 0 (HR: 0.53, 95% CI: 0.36–0.79), Child-Pugh class A (HR: 0.49, 95% CI: 0.34–0.71), lymph node metastasis present (HR: 0.54, 95% CI: 0.40–0.74), and distant metastasis absent (HR: 0.54, 95% CI: 0.38–0.75). The results of the above analysis are shown in **Figure 5**.

### 3.7 Sensitivity analyses

In the sensitivity analyses, a random-effects model was applied, and each study was systematically excluded in turn to evaluate the robustness of the prognostic role of hepatic arterial infusion chemotherapy (HAIC) compared to systemic chemotherapy in advanced intrahepatic cholangiocarcinoma (ICC). Specifically, sensitivity analyses were conducted for studies related to HAIC vs. Systemic Chemotherapy: Impact on Overall Survival in Advanced Intrahepatic Cholangiocarcinoma and HAIC vs. Systemic Chemotherapy: Impact on Progression-Free Survival in Advanced Intrahepatic Cholangiocarcinoma. The analyses demonstrated consistent results, further confirming the reliability of the findings and indicating that the conclusions are robust. Additionally, recalculations performed after excluding studies with smaller sample sizes or lower Newcastle-Ottawa Scale (NOS) scores produced consistent results, further supporting the reliability of the findings. These analyses suggest that the conclusions drawn from this meta-analysis are robust and not unduly influenced by any single dataset. Sensitivity analyses were conducted using StataMP 17 software (StataCorp. 2022. Stata Statistical Software: Release 17), and the results confirmed the robustness of the conclusions. Comprehensive details of the sensitivity analyses for each type of adjuvant therapy are provided in the supplementary materials. The results of the above analysis are shown in **Supplementary Figures 1**, **2**.

### 3.8 Publication bias

In studies comparing hepatic arterial infusion chemotherapy (HAIC) and systemic chemotherapy for overall survival in advanced intrahepatic cholangiocarcinoma, the symmetrical distribution of the funnel plots indicated no significant risk of publication bias. Additionally, Egger’s regression test confirmed this finding, demonstrating an insignificant presence of publication bias with a p-value of 0.937. The results of the above analysis are shown in **Supplementary Figures 3**, **4**.

In studies comparing hepatic arterial infusion chemotherapy (HAIC) and systemic chemotherapy for progression-free survival (PFS) in advanced intrahepatic cholangiocarcinoma, the symmetrical distribution of the funnel plots indicated no significant risk of publication bias. Furthermore, Egger’s regression test confirmed this finding, demonstrating an insignificant presence of publication bias with a p-value of 0.585. The results of the above analysis are shown in **Supplementary Figures 5**, **6**.

**Figure 6:**
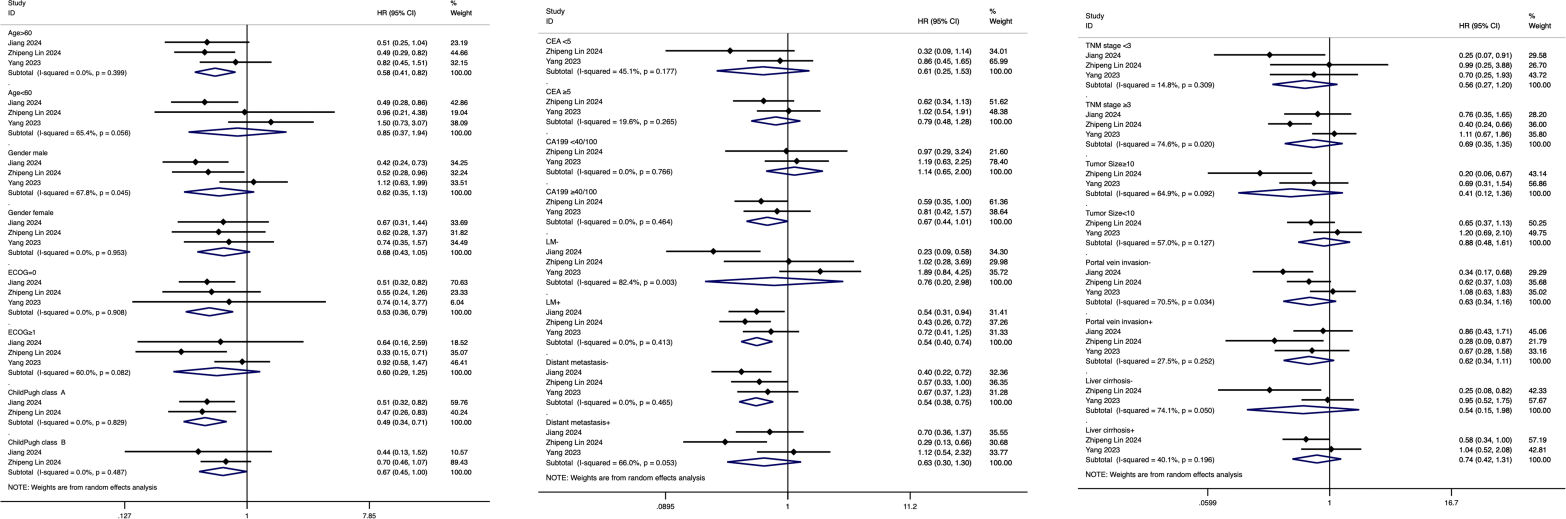
Figure abstract of hepatic arterial infusion chemotherapy in advanced intrahepatic cholangiocarcinoma.

As for Prognostic Factors Influencing Survival in Intrahepatic Cholangiocarcinoma Patients Undergoing HAIC Therapy and Prognostic Factors Influencing the Differences Between HAIC and Systemic Chemotherapy in Advanced Intrahepatic Cholangiocarcinoma Patients, no assessment of publication bias was performed due to the limited number of original studies focusing on individual factors. A figure abstract has been created to succinctly illustrate the main findings of this study (**Figure 6**).

## 4 Discussions

This meta-analysis reveals that hepatic arterial infusion chemotherapy (HAIC) demonstrates significant survival benefits over systemic chemotherapy in the treatment of advanced intrahepatic cholangiocarcinoma (ICC). HAIC was associated with improved overall survival (OS) and progression-free survival (PFS), with hazard ratios indicating a marked reduction in mortality and disease progression risk. Prognostic analyses identified factors such as ECOG performance status, CA 19–9 levels, and TNM stage as critical determinants of survival in ICC patients undergoing HAIC. Furthermore, subgroup evaluations suggest that specific patient characteristics, including older age, better liver function (Child-Pugh class A), and the absence of distant metastasis, may predict enhanced outcomes with HAIC compared to systemic chemotherapy. These findings underscore HAIC’s potential as an effective locoregional treatment strategy and highlight the importance of individualized therapeutic approaches in managing advanced ICC. To our knowledge, this is the first meta-analysis specifically comparing hepatic arterial infusion chemotherapy (HAIC) with systemic chemotherapy in intrahepatic cholangiocarcinoma (ICC).

An increasing body of evidence supports the therapeutic benefits of hepatic arterial infusion chemotherapy (HAIC). A review of nine studies, encompassing 478 patients with unresectable intrahepatic cholangiocarcinoma (iCCA) treated with hepatic arterial infusion chemotherapy (HAIC) using floxuridine, often in combination with systemic chemotherapy, demonstrated the efficacy of HAIC. The review reported a favorable overall survival (OS) rate, with a pooled 3-year OS of 39.5%. This outcome surpasses that observed with systemic chemotherapy in the ABC trials, where no patient survived beyond 3 years, underscoring the potential of HAIC as an effective treatment option for iCCA (28). In addition, Ghiringhelli et al. reported that the median OS of patients who received HAIC using gemcitabine and oxaliplatin as the second-line treatment was 20.3 months (29). A possible explanation is that hepatic arterial infusion chemotherapy (HAIC) achieves higher concentrations of chemotherapeutic agents in the liver compared to systemic chemotherapy (SC), enhancing its ability to control tumors in this region. The liver’s unique dual blood supply plays a critical role in this process. Specifically, the hepatic artery supplies nearly all the blood flow to the tumor, while the portal vein primarily supplies the non-neoplastic liver parenchyma. By preferentially delivering chemotherapeutic agents via the hepatic artery, HAIC more effectively targets and controls liver tumors (22). In addition to advanced intrahepatic cholangiocarcinoma (iCCA), hepatic arterial infusion (HAI) has demonstrated significant benefits in the treatment of extensive colorectal cancer liver metastases. Compared to systemic chemotherapy alone, HAI is associated with improved treatment responses, reduced toxicity, and a potential survival advantage, highlighting its efficacy in managing liver-dominant metastatic disease (20).

There is growing evidence that combining hepatic arterial infusion chemotherapy (HAIC) with other therapeutic strategies offers benefits for advanced intrahepatic cholangiocarcinoma (iCCA). For instance, patients treated with HAIC combined with lenvatinib and PD-(L)1 inhibitors (HLP) demonstrated significantly improved progression-free survival (PFS) and objective response rate (ORR) compared to those receiving systemic chemotherapy (SC), either with or without PD-(L)1 inhibitors (30, 31). While the overall survival (OS) in the HLP group was not statistically superior to the SCP group, it was notably better than the SC group. Widely endorsed in Asia as a treatment for advanced hepatocellular carcinoma (HCC), HAIC has also shown remarkable efficacy when combined with sorafenib or the combination of PD-1 inhibitors and lenvatinib (32). The observed benefits of HAIC in combination therapy may be attributed to synergistic mechanisms, including the ability of chemotherapy to induce tumor apoptosis through DNA damage and immunogenic cell death, thereby enhancing antitumor immune responses and boosting the effectiveness of immunotherapy (33). Additionally, small-molecule tyrosine kinase inhibitors, such as lenvatinib, improve the tumor microenvironment by targeting VEGFR1–3 and fibroblast growth factor receptor 1 (FGFR1), while regulating colony-stimulating factor 1 receptor (CSF1R) to reduce M2-type macrophage infiltration and suppress regulatory T cells (Tregs) (34). These effects weaken immunosuppressive responses, enhance the activity of PD-1 and PD-L1 inhibitors, and further promote robust immune responses, highlighting the potential of HAIC-based combination therapies in advanced iCCA.

Hepatic arterial infusion chemotherapy (HAIC) has not been directly assessed in a meta-analysis for safety and adverse events, but existing studies suggest that HAIC has certain advantages over systemic chemotherapy (SC) in these aspects. The target lesions in intrahepatic cholangiocarcinoma (iCCA) are predominantly located within the liver. Hepatic arterial infusion chemotherapy (HAIC) delivers chemotherapy drugs directly to the intrahepatic target lesion via the hepatic artery, leveraging the liver’s first-pass effect to significantly lower systemic drug levels. This approach reduces systemic toxicity and minimizes adverse reactions. In contrast, systemic chemotherapy is administered intravenously, requiring higher drug concentrations in the systemic circulation to achieve therapeutic effects at the target site. This can lead to increased toxicity, harm to multiple organ systems, and a higher likelihood of adverse events. A 2023 study found that the overall incidence of adverse events (AEs), including rash, vomiting, fatigue, leukopenia, anemia, and sensory neuropathy, was lower in patients receiving hepatic arterial infusion chemotherapy (HAIC) compared to those undergoing systemic chemotherapy (SC). Additionally, grade 3–4 AEs, such as hematologic toxicity and liver function damage, were less frequent in the HAIC group (22). LIN ET AL found similar results, the HAIC+L+P group experienced fewer TRAEs than the SC group (21). However, specific complications such as biliary toxicity and liver-related effects require vigilant management. The combination of HAIC with systemic agents, such as bevacizumab, has been explored. While such combinations showed efficacy, they sometimes led to increased biliary toxicity without significant improvements in outcomes, emphasizing the need for careful patient selection and monitoring (35). Future trials focusing on optimizing HAIC protocols and exploring its integration with systemic treatments are necessary to establish its broader applicability.

HAIC has shown promise in improving overall and progression-free survival compared to systemic chemotherapy in advanced intrahepatic cholangiocarcinoma (ICC). Its clinical application lies in leveraging these survival benefits to develop tailored treatment approaches for patients based on their individual prognostic profiles. This analysis highlights its potential role by identifying key prognostic factors and tailoring treatment strategies accordingly. Patients with unfavorable indicators—such as advanced TNM stage, multifocal or large tumors, elevated CA 19–9 or CEA levels, and ECOG =0—may require more intensive treatment regimens and closer follow-up. Conversely, those with localized disease, preserved liver function, and robust performance status may derive greater benefits from HAIC, allowing for more focused and potentially less invasive management plans. The findings also underscore the importance of subgroup analysis in clinical decision-making. For instance, patients aged >60, those with lymph node metastasis, or without distant metastases tend to achieve better outcomes with HAIC compared to systemic chemotherapy. Meanwhile, systemic chemotherapy may remain a viable option for patients with widespread disease or specific biomarker profiles where HAIC’s efficacy could be limited. This study offers valuable insights into the clinical application of HAIC by integrating prognostic factors to enhance personalized treatment strategies for advanced ICC. These results emphasize the need to balance treatment intensity with patient-specific characteristics to optimize outcomes while minimizing unnecessary interventions. Nonetheless, further well-designed, large-scale studies are essential to validate these conclusions and refine clinical protocols.

This meta-analysis is the first to explore the efficacy of hepatic arterial infusion chemotherapy (HAIC) compared to traditional systemic chemotherapy in intrahepatic cholangiocarcinoma (ICC), providing a meaningful addition to the existing body of research. However, several limitations should be noted. The small number of studies included in this analysis increases the potential for heterogeneity, which may limit the generalizability of the findings. Additionally, the restricted data set hampers the ability to conduct more detailed subgroup analyses, which could provide deeper insights into specific patient populations or treatment scenarios. Another limitation is the absence of a comprehensive evaluation of important clinical factors, such as patient quality of life, toxicity management, and treatment costs, which are essential for informed clinical decision-making. Furthermore, variations in HAIC protocols across the included studies, including differences in treatment regimens, may have introduced heterogeneity, potentially affecting the reliability and consistency of the results.

## 5 Conclusion

This meta-analysis indicates that hepatic arterial infusion chemotherapy may offer survival benefits over systemic chemotherapy in advanced intrahepatic cholangiocarcinoma, with potential improvements in overall survival and progression-free survival. Key prognostic factors influencing treatment outcomes include gender, ECOG performance status, elevated CA 19–9 levels, advanced TNM stage, and poorer liver function. Future large-scale, well-designed studies are essential to validate these findings, optimize protocols, and enhance clinical decision-making for advanced intrahepatic cholangiocarcinoma management.

## Data Availability

The original contributions presented in the study are included in the article/**Supplementary Material**. Further inquiries can be directed to the corresponding author/s.

## Supplementary material

Supplementary Figure 1 Sensitivity analysis for overall survival comparing HAIC and systemic chemotherapy in advanced ICC.

Supplementary Figure 2 Sensitivity analysis for progression-free survival comparing HAIC and systemic chemotherapy in advanced ICC.

Supplementary Figure 3 Funnel plot for publication bias in overall survival analysis between HAIC and systemic chemotherapy.

Supplementary Figure 4 Funnel plot for publication bias in progression-free survival analysis between HAIC and systemic chemotherapy.

Supplementary Figure 5 Egger’s test results for publication bias in overall survival analysis.

Supplementary Figure 6 Egger’s test results for publication bias in progression-free survival analysis.

Supplementary Table 1 Newcastle-Ottawa Scale (NOS) quality assessment scores for the included studies. The table provides a breakdown of scores across three domains: selection, comparability, and outcome, demonstrating the high quality of the majority of included studies.
