# Supplementary figures and images for: Hepatic arterial infusion chemotherapy versus systemic chemotherapy for advanced intrahepatic cholangiocarcinoma: a meta-analysis of survival outcomes
Source: Front Immunol. 2025 Jul 16;16:1640970. doi: 10.3389/fimmu.2025.1640970 (PMC12309412; doi:10.3389/fimmu.2025.1640970)

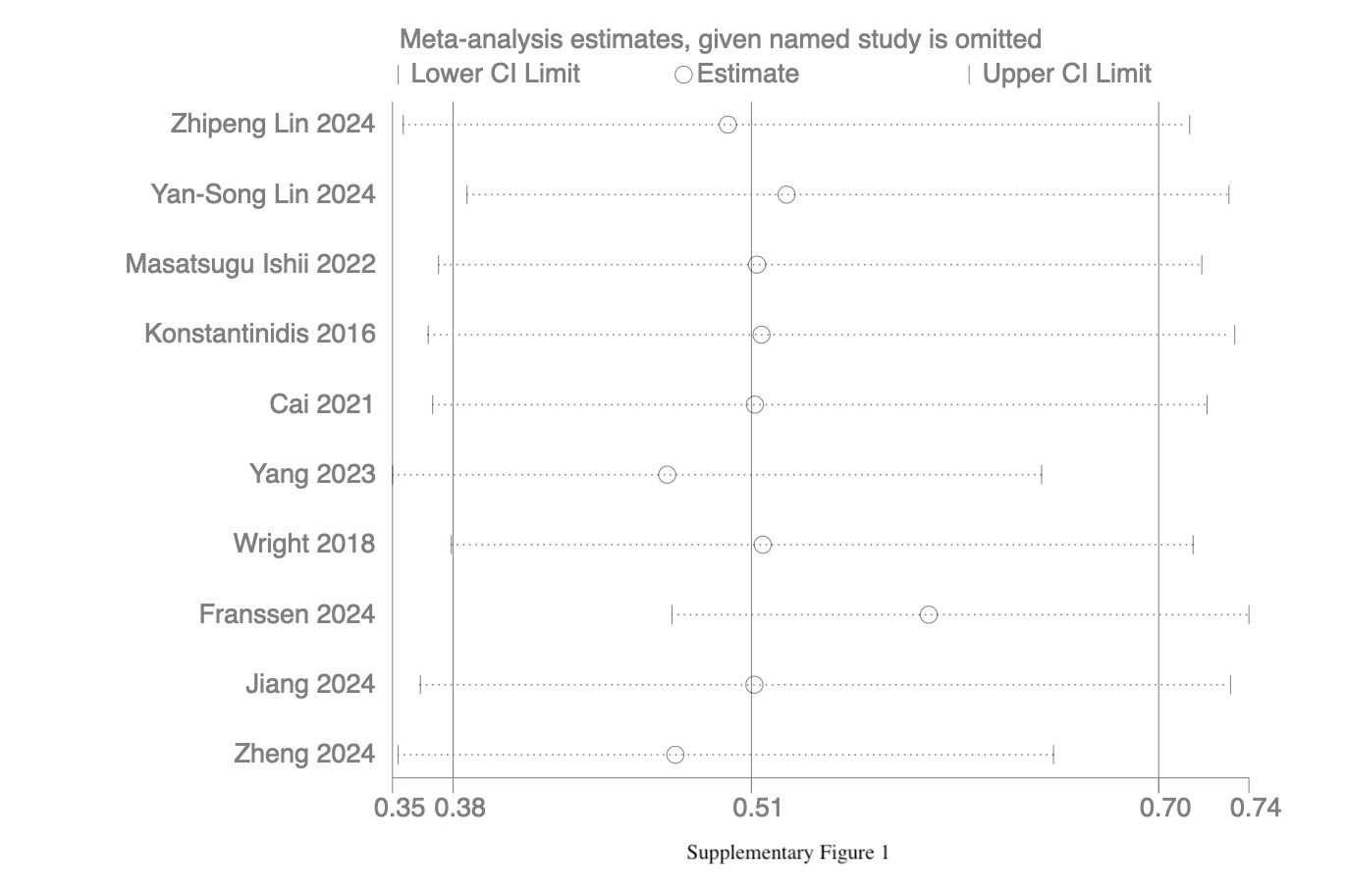

Supplement: Supplementary Figure 1 — Sensitivity analysis for overall survival comparing HAIC and systemic chemotherapy in advanced ICC. [file Image1.jpeg]

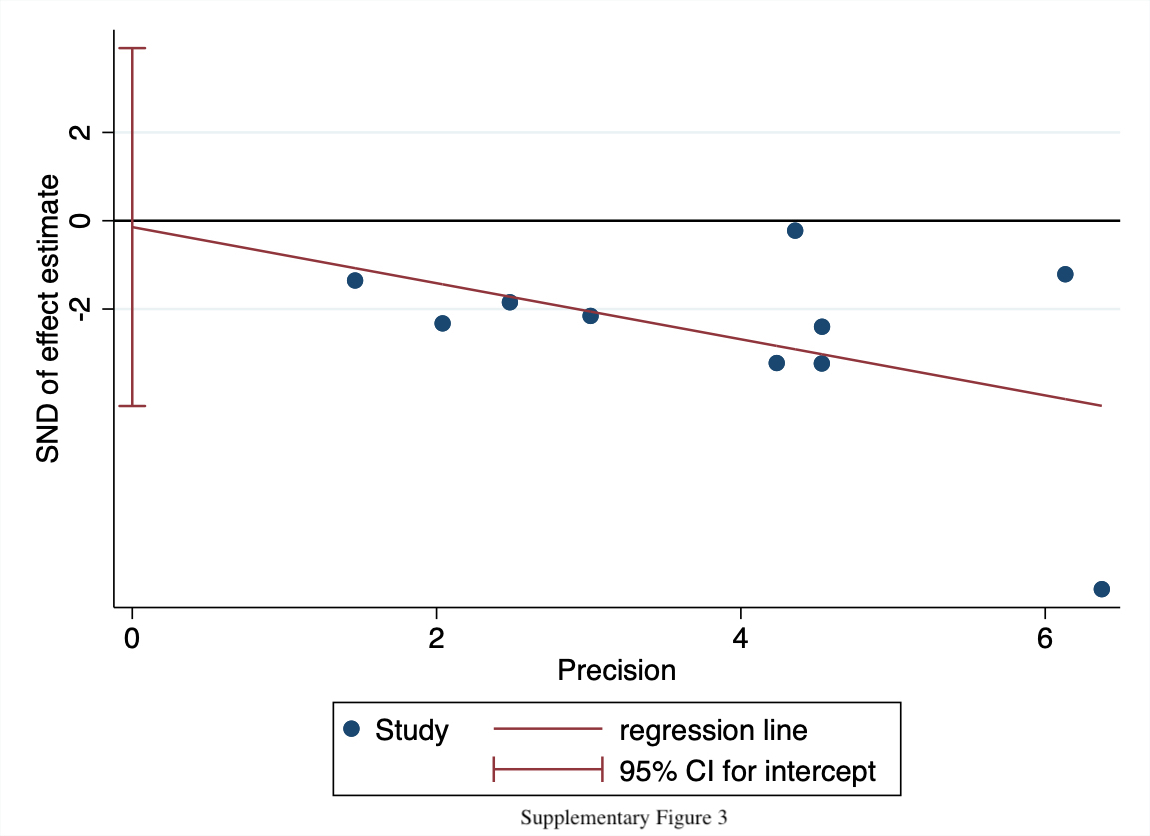

Supplement: Supplementary Figure 2 — Sensitivity analysis for progression-free survival comparing HAIC and systemic chemotherapy in advanced ICC. [file Image2.jpeg]

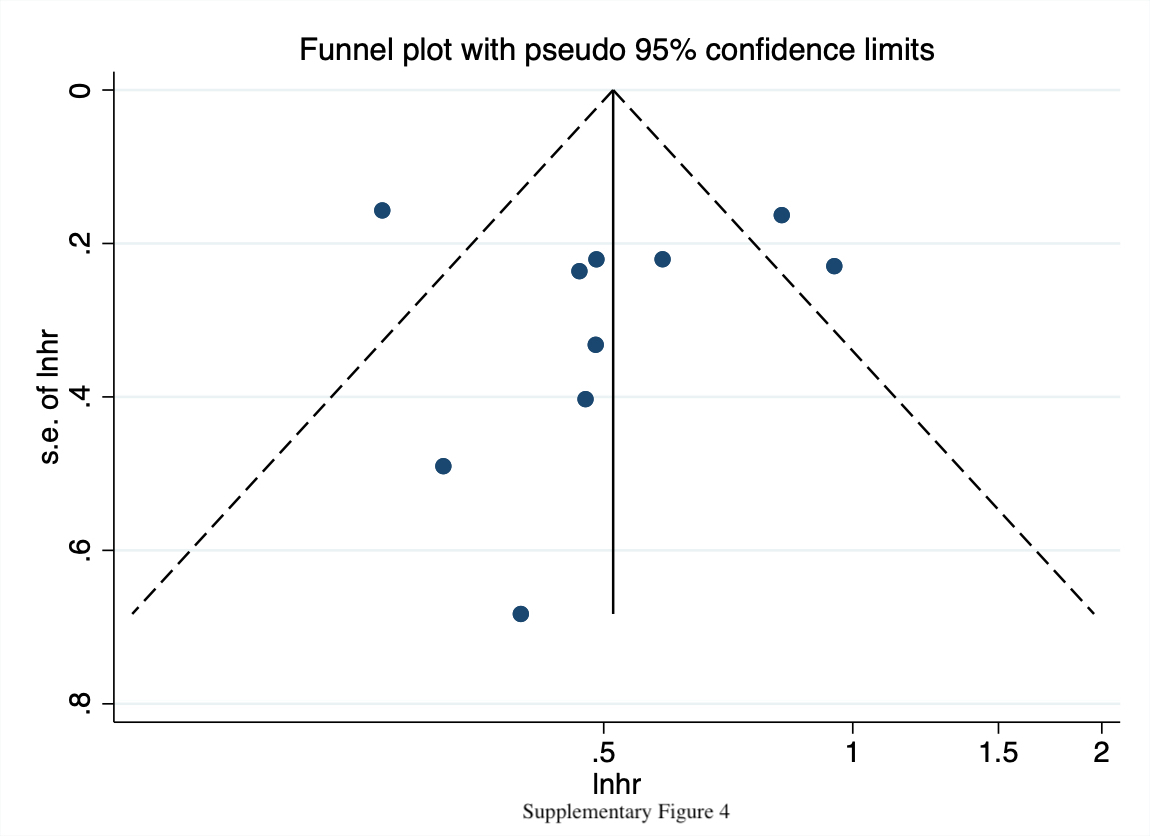

Supplement: Supplementary Figure 3 — Funnel plot for publication bias in overall survival analysis between HAIC and systemic chemotherapy. [file Image3.jpeg]

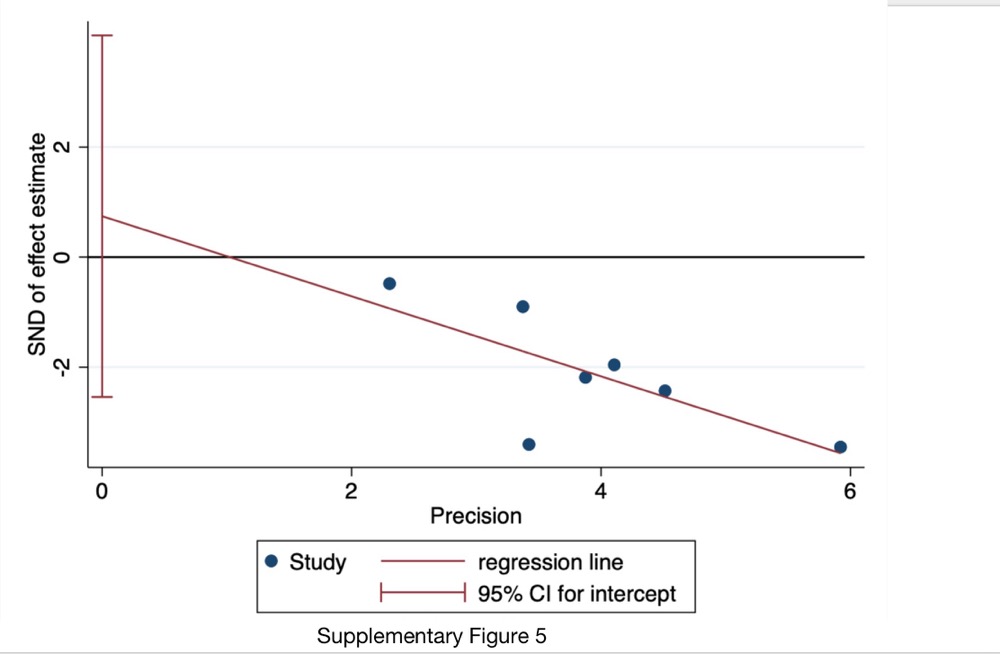

Supplement: Supplementary Figure 4 — Funnel plot for publication bias in progression-free survival analysis between HAIC and systemic chemotherapy. [file Image4.jpeg]

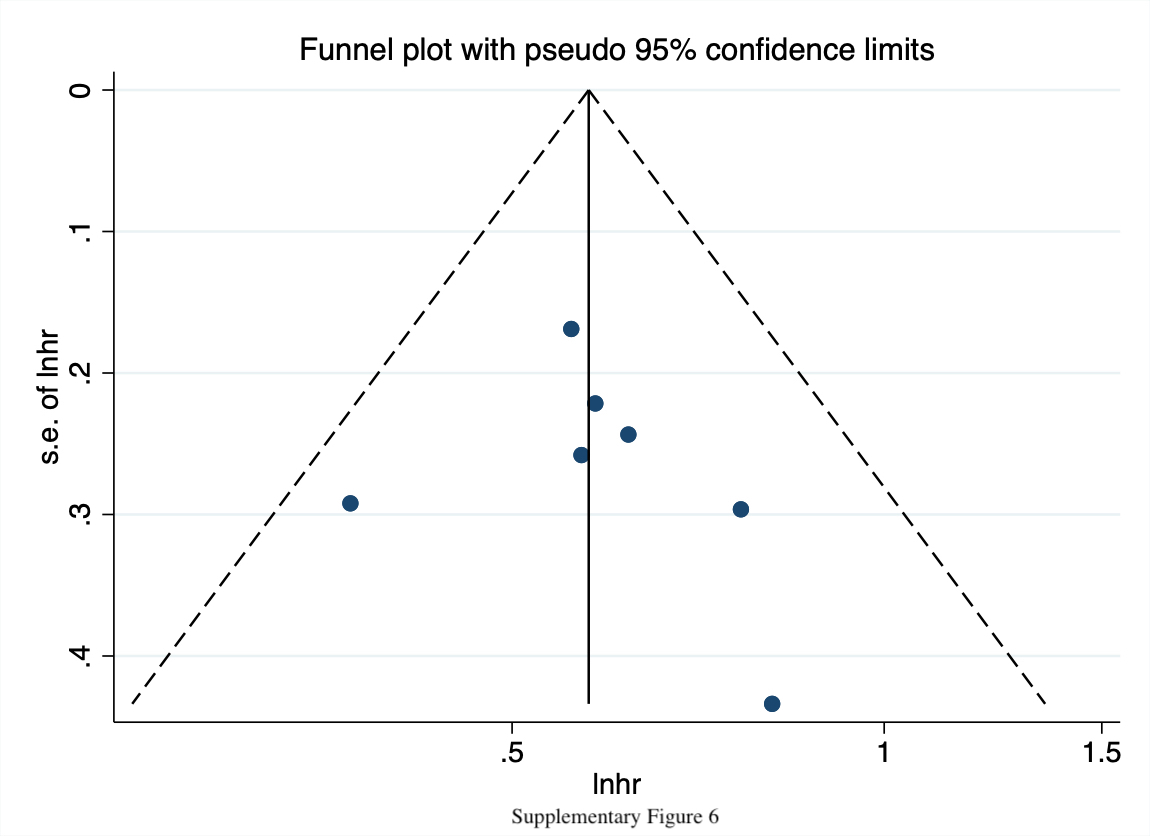

Supplement: Supplementary Figure 5 — Egger’s test results for publication bias in overall survival analysis. [file Image5.jpeg]
