# Supplementary material for: Hepatic arterial infusion chemotherapy versus systemic chemotherapy for advanced intrahepatic cholangiocarcinoma: a meta-analysis of survival outcomes
Source: Front Immunol. 2025 Jul 16;16:1640970. doi: 10.3389/fimmu.2025.1640970 (PMC12309412; doi:10.3389/fimmu.2025.1640970)
Supplement: Supplementary Table 1 — Newcastle-Ottawa Scale (NOS) quality assessment scores for the included studies. The table provides a breakdown of scores across three domains: selection, comparability, and outcome, demonstrating the high quality of the majority of included studies. [file Table1.docx]

| study | selection | | | | comparability | Outcome | | | NOS score |
| --- | --- | --- | --- | --- | --- | --- | --- | --- | --- |
|  | Representativeness of the exposed cohort | Selection of the non-exposed cohort | Ascertainment of exposure | Demonstration that outcome of interest was not present at start of study | Comparability of cohorts based on the design or analysis | Assessment of outcome | Was follow-up long enough for outcomes to occur | Adequacy of follow up of cohorts |  |
| **Zhipeng Lin 2024** | ★ | ★ | ★ | ★ | ★ |  | ★ | ★ | 7 |
| **Yan‐Song Lin 2024** | ★ | ★ | ★ | ★ | ★★ | ★ | ★ | ★ | 9 |
| **Masatsugu Ishii 2022** | ★ | ★ | ★ | ★ | ★ | ★ | ★ | ★ | 8 |
| **Konstantinidis 2016** | ★ | ★ | ★ | ★ | ★ | ★ | ★ | ★ | 8 |
| **Cai 2021** | ★ | ★ | ★ | ★ | ★ | ★ | ★ |  | 7 |
| **Yang 2023** | ★ | ★ | ★ | ★ | ★ | ★ |  | ★ | 7 |
| **Wright 2018** | ★ | ★ | ★ | ★ | ★ | ★ | ★ | ★ | 8 |
| **Franssen 2024** | ★ | ★ | ★ | ★ | ★ | ★ | ★ | ★ | 8 |
| **Jiang 2024** | ★ | ★ | ★ | ★ | ★ | ★ | ★ | ★ | 8 |
| **Zheng 2024** | ★ | ★ | ★ | ★ | ★ | ★ | ★ | ★ | 8 |

Supplementary Table 1: results of quality assessment using the Newcastle-Ottawa Scale for cohort Studies.

Legend: risk of bias summary and graph showing authors’ judements about each risk of bias domain for observational studies using the NOS tool
